# Case report: A rare occurrence of triple malignancy of the stomach, rectum and liver in a single patient

**DOI:** 10.3389/fonc.2022.945689

**Published:** 2022-09-20

**Authors:** Cancan Jin, Jiangnan Hu, Kirthikaa Balapattabi, Brian Wang, Sizhe Hu, Kangyi Wang, Liangbin Fu, Xiaokang Zhao, Feng Qian, Zhen Wang

**Affiliations:** ^1^ Department of Oncology, Dongyang Hospital of Wenzhou Medical University, Dongyang, China; ^2^ Department of Dermatology and Cancer Center, Medical College of Wisconsin, Milwaukee, WI, United States; ^3^ Department of Physiology, Medical College of Wisconsin, Milwaukee, WI, United States; ^4^ Pathnova Laboratories Pte. Ltd., Singapore, Singapore; ^5^ Department of Colorectal Surgery, The Second Affiliated Hospital of Harbin Medical University, Harbin, China

**Keywords:** gastric carcinoma, hepatocellular carcinoma, rectal carcinoma, surgery, immune checkpoint inhibitors

## Abstract

Malignant tumors of the digestive system are common worldwide; however, it is extremely rare for more than two malignancies to occur simultaneously. Here, we report a case with a triple malignancy of the digestive system, including gastric, rectal, and hepatic tumors. The patient underwent surgical resection of three tumors followed by chemotherapy. Negative image-based screenings and the absence of serum tumor biomarkers elevation were found at 2.5 years after the surgery, indicating the absence of recurrence and metastasis of cancers.

## Background

Cancer is a leading cause of death worldwide and remains the major obstructive factor for a rise in global life expectancy. In 2020, the World Health Organization reported approximately 10 million cancer deaths worldwide. Colorectal and stomach cancer are the third and fourth leading causes of death, followed by liver cancer. According to a study done by the National Cancer Institute in 2011, the number of patients with multiple primary cancers is increasing dramatically (1).

In this report, we present a unique and important case of a patient with triple malignancy of the stomach, liver, and colorectum. The gold standard criteria outlined by Warren and Gates (2) was adapted for patient diagnosis: (i) Each tumor must have a clear image and histological confirmation of malignancy; (ii) Each tumor must be topographically distinct and separated by a healthy mucosa (at least 2 cm); (iii) The lesions must be primary, not metastases of each other. Fortunately, diagnostic equipment such as PET/CT has advanced to such a level that enables identification of multiple synchronous malignancies occurring in various organs like the thyroid, breast, head, neck, and others (3, 4).

Based on the diagnosis, the patient underwent surgical removal of the triple tumors and was given chemotherapy thereafter. Most encouragingly, no signs of recurrence or metastases have occurred so far. As triple primary malignancies are rare, no large case studies describing the characteristics and outcomes in such patients have been reported (see **Table 1**). The treatment plan for patients with multiple tumors presents many challenges, and a multidisciplinary approach with tailored decision-making for the individual patient helps to achieve optimum outcomes. It is our hope that detailing this rare clinical case including our strategies for patient care and management can serve as a reference for any similar future clinical cases.

**Table 1:** Summary of cases with multiple primary tumors (three and above) reported in literature.

Author, Year	Age/Sex	Tumors Reported
Kim et al. (5), 2013	73/F	Thyroid carcinoma/Breast carcinoma/Pancreatic carcinoma/Gastrointestinal stromal tumor
Grace et al. (6), 2015	70/M	Glioblastoma/Schwannoma/Neuroendocrine tumor/Adenoma
Meeks et al. (7), 2016	95/F	Adenocarcinoma/Adenoma/Neuroendocrine tumor/Schwann cell hamartoma
Elec et al. (8), 2017	78/M	Prostate adenocarcinoma/Clear cell renal carcinoma/Papillary renal carcinoma/Bladder cancer
Nanashima et al. (9), 2017	67/M	Stomach/Sigmoid colon/Rectum/Pancreas carcinomas
Kataoka et al. (10), 2017	72/M	Pharyngeal squamous cell carcinoma/Esophageal squamous cell carcinoma/Esophageal adenocarcinoma
Wang et al. (11), 2019	56/F	Cervix/Endometrium/Ovary/Stomach carcinomas
Tanaka et al. (12), 2020	73/M	Stomach/Intrahepatic Bile Duct/Prostate carcinomas
Albaqmi et al. (13), 2020	63/M	Stomach/Colon/Kidney carcinomas
Takada et al. (14), 2020	89/M	Stomach/Lung/Breast carcinomas
Takada et al. (15), 2017	71/F	Breast/Duodenal/Lung carcinomas
Katz et al. (16), 2017	48/F	Colon/Ovary/Adrenal gland carcinomas
Sauri et al. (17), 2021	61/M	Stomach adenocarcinoma/Esophageal squamous cell carcinoma/Colon adenocarcinoma

## Case presentation

**Figure 1:**
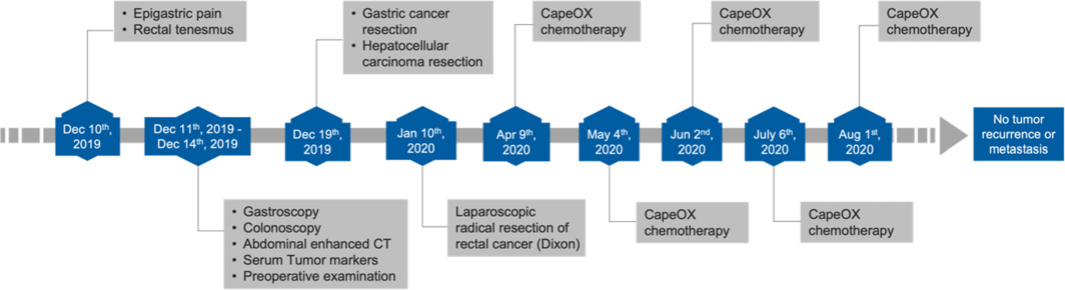
Timeline outlining clinical events.

**Figure 2:**
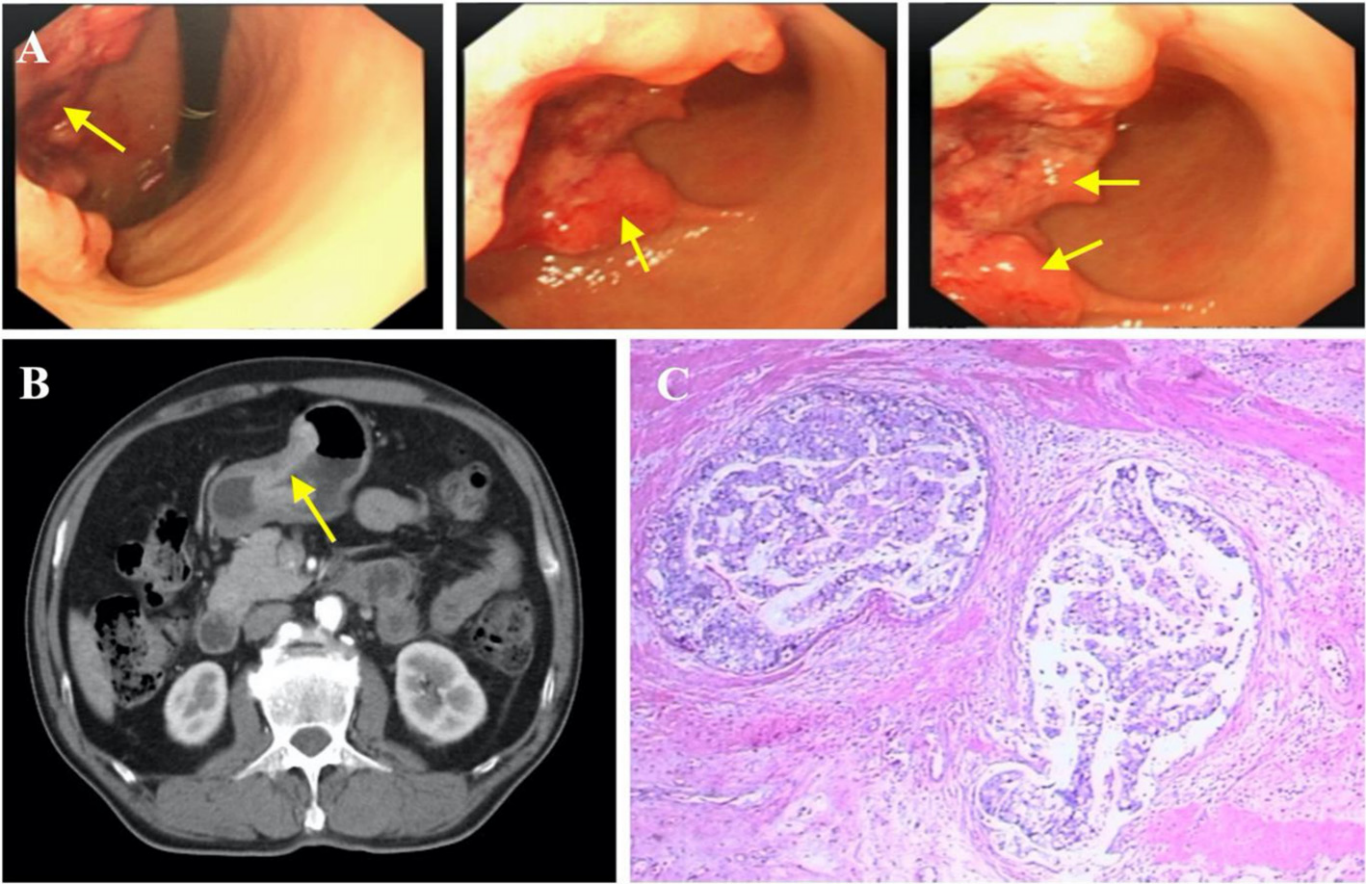
Diagnosis of gastric carcinoma. **(A)** Endoscopic images showing gastric cancer. **(B)** Gastric cancer (indicated by yellow arrow), specific enhancement pattern on multiphasic contrast-enhanced computed tomography (CT). **(C)** Representative histopathological image of gastric cancer.

A 64-year-old male patient presented with epigastric pain for half a month and the sensation of rectal tenesmus for more than three months. The timeline of events is presented **Figure 1**. According to the Chinese Society of Clinical Oncology (CSCO) guidelines, a gastroscopy was first performed where a tumor was found at the angular notch of the stomach (**Figure 2A**). Elevated alpha-fetoprotein (AFP) and carcinoembryonic antigen (CEA) serum levels were at 34.25 ng/ml and 30.11 ng/ml, respectively. There was also no indication of infection due to Hepatitis A, B, and C viruses.

**Figure 3:**
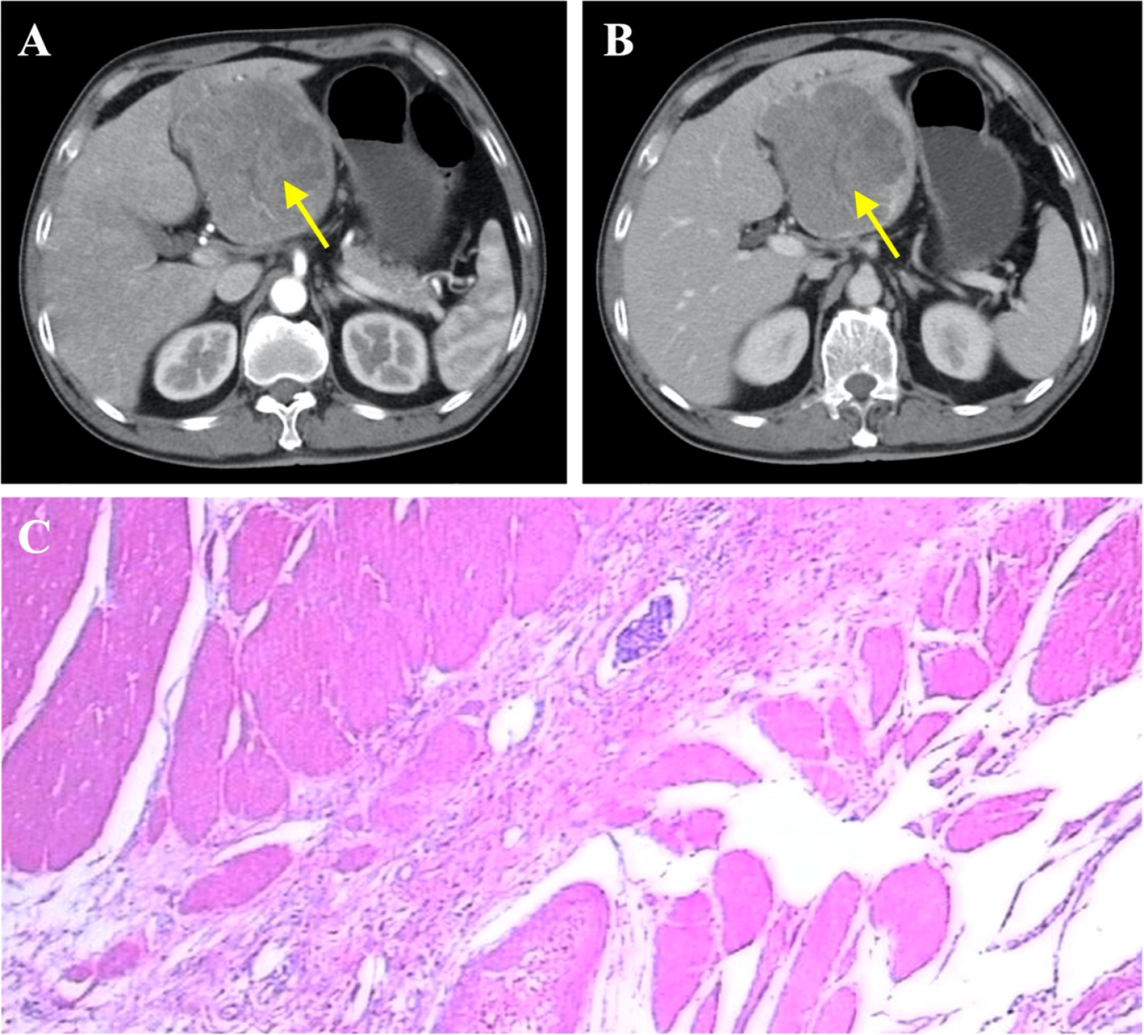
Diagnosis of primary liver cancer. **(A)** Liver cancer seen on contrast-enhanced CT (arterial phase), indicated by yellow arrow. **(B)** Liver cancer seen on contrast-enhanced CT (venous phase), indicated by arrow. **(C)** Representative histopathological image of liver cancer.

**Figure 4:**
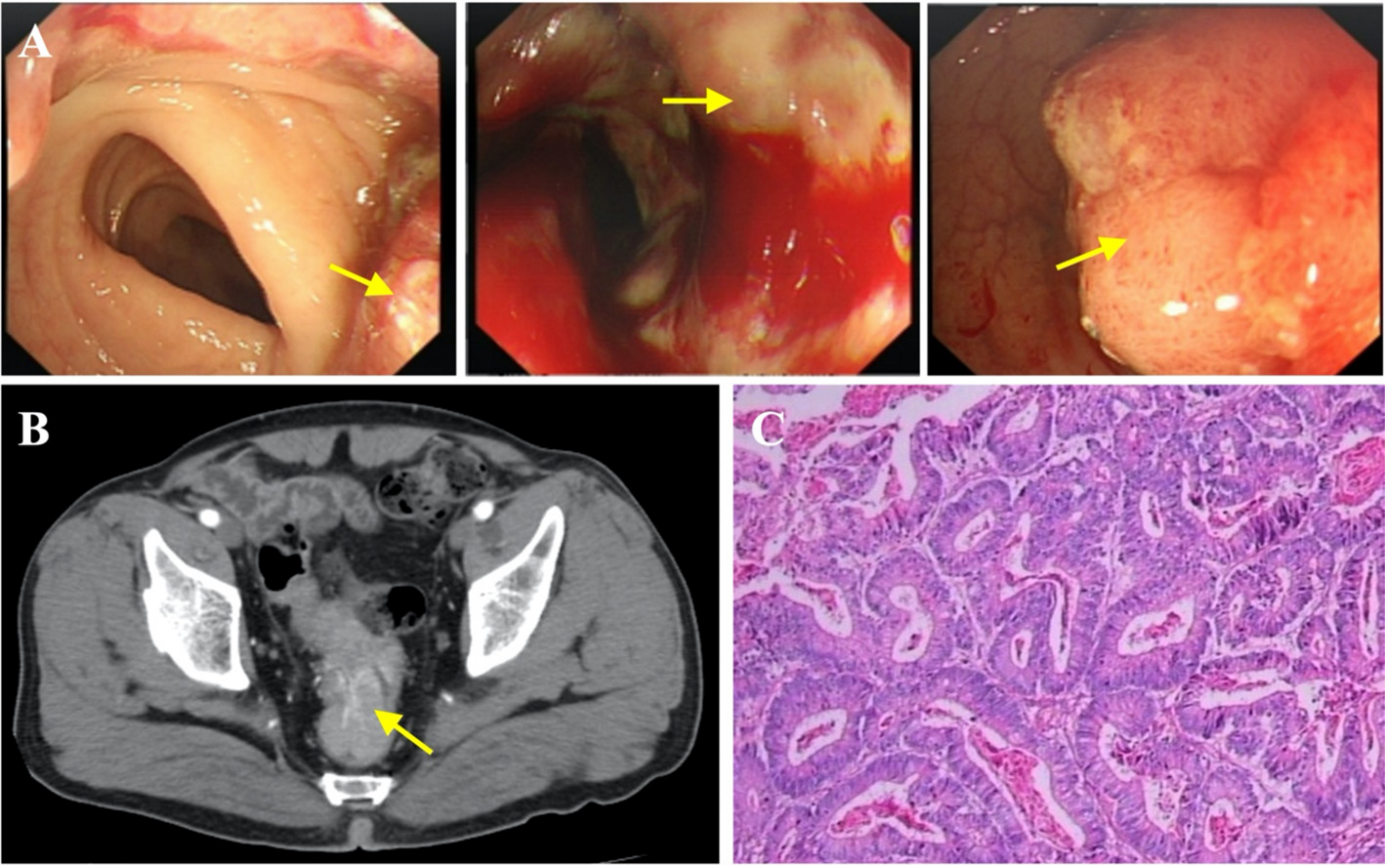
Diagnosis of rectal carcinoma. **(A)** Endoscopic images indicating rectal carcinoma (yellow arrow). **(B)** Contrast-enhanced CT showing rectal cancer. **(C)** Representative histopathological image of rectal carcinoma.

To further determine the cause of increased AFP and CEA levels, contrast-enhanced computed tomography (CT) was performed that showed a primary tumor in the left lobe of the liver (**Figures 3A, B**), with possible existing carcinomas in the stomach (**Figure 2B**) and the rectum (**Figure 4B**). To assess the mass in the rectum, a colonoscopy revealed an erythematous mass located at roughly 10 cm away from the pectinate line. The mass had an uneven and brittle surface that was eroded, and also occupied about two-thirds of the intestinal lumen, resulting in colon stenosis. A biopsy indicated that the mass was a rectal adenocarcinoma which was prone to bleeding (**Figure 4A**).

A multidisciplinary team comprising a gastrointestinal surgeon, an oncology surgeon, a radiologist, a histopathologist, and an oncologist was then convened. Based on the findings, the team agreed that no tumors were found in other organs and that the patient could tolerate surgical treatment. Although most tumors are resectable, the simultaneous resection of multiple tumors poses a high risk. A decision was then reached to perform tumor resection in two stages.

The first stage involved radical gastrectomy of the stomach carcinoma, a left hemihepatectomy and cholecystectomy. During the operation, the samples from the lesion sites of stomach and liver were biopsied, both pathological examinations show malignant tumors (**Figures 2C**, **3C**). The pathological analysis of the gastric tumor revealed an erythematous mass with an irregular border located at the anterior wall of the gastric antrum (size: 5 × 5 × 1 cm) and suggested an ulcer uplifted type of moderately differentiated adenocarcinoma that was immersed under the serous membrane. Lymphovascular emboli were present and perineural invasion was absent. Twenty-one surrounding lymph nodes around the stomach were removed, six of which were metastatic. The TNM (Tumor Node Metastasis) stage of the gastric cancer was T_3_N_3a_M_0_ (stage IIIB). Histological analysis of the hemihepatectomy showed chronic cholecystitis and poorly differentiated adenocarcinoma (size: 10 × 10 cm) with a negative margin of resection. Immunohistochemistry interrogation revealed that it was CerbB-2 (-), P53 (-), Ki-67 (approximately 70% +), AFP (-), Hepatocyte (-), CD34 (-), CK20 (-), CDX2 (+), CK8 (-), CK19 (-), and Gly-3 (-). The liver mass was also confirmed to be malignant. In addition, based on the CT images, we found that the liver tumor was isolated and located in the left lobe with no signs of vascular invasion. Therefore, surgical resection was the primary treatment since the tumor was isolated and complete resection was feasible (18). However, we acknowledge that an in-depth pathological study could have been done to further identify the nature of the cancer.

The second stage was a laparoscopic radical resection of the rectal adenocarcinoma (Dixon). Pathological analysis showed a rectal ulcerative type of moderately differentiated adenocarcinoma (size: 7 × 4.5 × 1.5 cm) infiltrating the outer membrane with the presence of lymphovascular emboli and absence of perineural invasion (**Figure 4C**). All nineteen lymph nodes were surgically removed and there were no signs of lymph node metastasis. The TNM stage of the rectal cancer was T_3_N_0_M_0_ (stage IIA).

Following the two-stage surgery, a combination chemotherapy with CapeOX (Capecitabine and Oxaliplatin) was given once a month for four consecutive months (**Figure 1**). Follow-up abdominal enhanced CT scan was done every 6 months and showed no tumor recurrence. In the most recent abdominal enhanced CT scan performed on May 26^th^, 2022, no tumor recurrence was indicated. AFP and CEA levels also declined to normal values postoperatively.

## Discussion and conclusion

Here, we describe a triple primary malignancy in a patient who subsequently had successful treatment. The patient had no prior medical history of any serious illness. The patient, whose father died of pancreatic cancer in his 70s, was advised to undergo a genetic evaluation before surgery, but the patient refused. Considering available evidence for the etiology of multiple cancers, it is likely to be multifactorial with possible influences from the environment with likely minimal genetic influence. The patient had a remote smoking history (quit smoking 18 years ago), smoking about sixty cigarettes daily for twenty years. The patient also had a history of alcohol intake; ~250 ml distilled spirits daily for forty years. The occurrence of multiple tumors could be highly related to his living environment and lifestyle habits. When the patient came to the hospital, three tumors were diagnosed at the same time, so the sequence of tumor incidence was not clear.

Although simultaneous occurrence of multiple gastrointestinal tumors has been reported (**Table 1**), it is still an extremely rare phenomenon seen in less than 0.5% of patients with cancer (19–22). One problem presented by multiple primary tumors is the risk that they could have been metastatic in nature, and thus change oncosurgical management to include preoperative chemotherapy instead. To investigate this, we evaluated the nature of the liver tumors as the liver is known to be one of the most common sites for cancer metastasis and also due to its proximity to the GI tract. Abdominal CT scans show that the liver tumors present with an exophytic growth pattern and a necrotic core at the lesion site. Based on our team’s collective experience, this was diagnosed as primary liver cancer. In addition, postoperative immunohistochemical analysis of the liver tumors revealed that they were all negative for AFP, Hep and Gly-3 markers, so the diagnosis of hepatocellular carcinoma was excluded. Moreover, the liver cancer cells showed no similarities when compared with the ones from the GI tumors. Taken together, we concluded that the liver tumors were primary in nature and had not metastasized from the gastric carcinoma.

The treatment of multiple primary cancers is still a challenge because of the lack of harmonized guidelines. However, surgical treatment is the standard of care for solid tumors in their early and advanced stages. For our patient, all tumors were resectable, and a long-term survival was expected with radical resection. Given that the surgical resections of the gastric and rectal tumors were significantly invasive, other additional treatments such as chemotherapy and radiotherapy were needed to improve the surveillance. According to the 2021 CSCO guidelines for the diagnosis and treatment of gastric cancer, the CapeOX and SOX (Tegafur Gimeracil and Oteracil Porassium Capsule and Oxaliplatin) were recommended for postoperative adjuvant chemotherapy for stage IIIB gastric cancer. The rectal tumor was located at the rectosigmoid junction and invaded the serosa, but the surgically removed lymph nodes showed no infiltration of tumor cells. Histology of the dissected colonic tumor suggested adventitia infiltration, which suggests a high-risk factor for metastasis. Importantly, CapeOX chemotherapy can also be used for managing colorectal cancer (23). In addition, our clinical team proposed radiotherapy to the patient based on the stage of gastric cancer (IIIB), but he declined due to financial difficulties and the risk of adverse effects. Eventually, as reported (**Figure 1**), the patient was given CapeOX chemotherapy five times following CSCO’s guidelines. In the course of chemotherapy, the patient developed myelosuppression and was unable to tolerate the side effects which led to the cessation of chemotherapy. The reference value of this case could be greatly increased if the entire chemotherapy was completed on time and in a standardized manner (for six consecutive months at a frequency of every three weeks). However, it has been 2.5 years since the patient was diagnosed with triple primary cancers and the latest follow-up examination results showed no signs of tumor recurrence or metastasis.

Recently, immune checkpoint inhibitors have been actively introduced into the management of gastrointestinal tumors, and their contribution, either as monotherapy or in combination is meaningful. Along with the development of precision medicine, immune checkpoint inhibitors will play a major life saving role in the treatment of gastrointestinal tumors. For our patient, we also proposed genetic testing for immunotherapy suitability, but he declined citing financial difficulty and personal reasons. In addition, HER-2 (Human Epidermal Growth Factor Receptor 2) expression was negative in the tumor biopsy, so targeted therapy with Herceptin was not applicable in this case (18).

We acknowledge that there were some shortcomings in our patient management since this is our first time experiencing a case of multiple synchronous tumors. The first being the irregularity of chemotherapy which could have affected full therapeutic benefits to be gained by the patient, but this factor was shown to be non-crucial since the patient is still in complete remission after 2.5 years. The second being the lack of relevant genetic tests conducted due to the patient’s refusal, which immediately excluded him from potential immunotherapy and exposed him to higher risks due to invasive surgical procedures. Lastly, our lack of clinical experience could have made the treatment flawed. The fact that we did not first distinguish whether the liver tumor originated from the gastrointestinal tumor created an uncertainty in patient management.

In conclusion, a few lessons can be gleaned from our experience. In the case of multiple primary synchronous tumors, clinicians should evaluate the nature of these tumors i.e., primary or metastasized, as this determines the appropriate oncosurgical plan for the patient. If the cancer has metastasized, neoadjuvant chemotherapy may be considered. Relevant genetic testing can also be subsidized by the healthcare institution or government and made available for those who present as candidates for suitable immunotherapy, which has shown great promise for GI tumors. Finally, a multidisciplinary approach to patient management has proven to be beneficial in that most aspects were covered to devise the best possible treatment plan.

## Data availability statement

The original contributions presented in the study are included in the article. Further inquiries can be directed to the corresponding authors.

## Ethics statement

The studies involving human participants were reviewed and approved by Dongyang Hospital of Wenzhou Medical University. The patients/participants provided their written informed consent to participate in this study.

## Author contributions

CJ, JH, and ZW conceived and drafted the manuscript. KB, BW, SH, KW, LF, XZ, and FQ reviewed and edited the manuscript. All authors read and approved the final version of the manuscript.

## Funding

This work was supported by Dongyang Hospital of Wenzhou Medical University and the Second Affiliated Hospital of Harbin Medical University.

## Conflict of interest

Author BW was employed by Pathnova Laboratories Pte. Ltd.

The remaining authors declare that the research was conducted in the absence of any commercial or financial relationships that could be construed as a potential conflict of interest.

## Publisher’s note

All claims expressed in this article are solely those of the authors and do not necessarily represent those of their affiliated organizations, or those of the publisher, the editors and the reviewers. Any product that may be evaluated in this article, or claim that may be made by its manufacturer, is not guaranteed or endorsed by the publisher.

## Abbreviations

AFP, Alpha-fetoprotein; CapeOX, Capecitabine and Oxaliplatin; CEA, Carcinoembryonic Antigen; CSCO, Chinese Society of Clinical Oncology; HER-2, Human Epidermal Growth Factor Receptor 2; TNM, Tumor Node Metastasis.
